# Effects of supplementation with *Bifidobacterium animalis* subsp. *lactis* CECT 8145—in live probiotic and heat-treated postbiotic form—on fecal metabolites, fecal microbiota, blood metabolites and systemic biomarkers of oxidative stress and inflammation, and white blood cell gene expression of adult cats

**DOI:** 10.1093/jas/skaf355

**Published:** 2025-10-21

**Authors:** Sergio Miranda de Souza Junior, Empar Chenoll, Adrian Howard-Varona, Araceli Lamelas, Juan F Martinez-Blanch, Gary M Davenport, Sophie Nixon, Fei He, Maria R C de Godoy

**Affiliations:** Division of Nutritional Sciences, University of Illinois, Urbana, IL 61801; ADM Biopolis, University of Valencia Science Park (Parc Científic de la Universitat de València, Valencia, 46980, Spain; ADM Biopolis, University of Valencia Science Park (Parc Científic de la Universitat de València, Valencia, 46980, Spain; ADM Biopolis, University of Valencia Science Park (Parc Científic de la Universitat de València, Valencia, 46980, Spain; ADM Biopolis, University of Valencia Science Park (Parc Científic de la Universitat de València, Valencia, 46980, Spain; ADM Biopolis, University of Valencia Science Park (Parc Científic de la Universitat de València, Valencia, 46980, Spain; ADM Health & Wellness, Lopen Head, Somerset, UK; Department of Animal Sciences, University of Illinois, Urbana, IL 61801; Division of Nutritional Sciences, University of Illinois, Urbana, IL 61801; Department of Animal Sciences, University of Illinois, Urbana, IL 61801

**Keywords:** biotics, butyrate, cytokines, feline, microbiome

## Abstract

Probiotics and postbiotics have been linked with enhancement of gastrointestinal health. This study aimed to evaluate the longitudinal effects of daily supplementation of a pro- and a postbiotic on metabolic health, blood metabolites and systemic biomarkers of oxidative stress and inflammation, fecal fermentative end-products, the gastrointestinal microbiota and white blood cell gene expression of adult cats. The study was a double-blind parallel randomized design with 36 cats divided into 3 groups. Animals were fed a standard extruded commercial diet. The groups were control diet + placebo (maltodextrin carrier) (CON), control diet + probiotic (*Bifidobacterium animalis* subsp*. lactis* CECT 8145; daily dosage: 10^10^ CFU/day) (PRO), and control diet + postbiotic (Heat-treated *Bifidobacterium animalis* subsp*. lactis* CECT 8145) 1x10^10^ cells/day (POST). Longitudinal analyses were performed every 30 d for a total of 90 d. There was no observed effect on body weight (*P* > 0.05). At a species level, cats in the PRO group had an increased abundance of *Bifidobacterium animalis* over time (*P* < 0.05). Over time, CAZy genes between the PRO and POST groups presented similar output when compared to the CON group. There was no treatment by day effect of serum cytokines and chemokines (*P* > 0.05). Overall, the supplementation of the probiotic and postbiotic was safe and well tolerated during the supplementation period, showing similar physiological responses in this population of adult cats.

## Teaser

Dive into innovative research to understand the promising effects of probiotics and postbiotics on adult cat health. Studying how bioactive agents modulate gut microbiota composition, fecal metabolite profiles, and systemic inflammatory markers, supporting gastrointestinal health and the immune system. Step into the forefront of feline nutrition research in this article.

## Introduction

Probiotics are defined as live microorganisms that when ingested offer the host health benefits (Hill et al., 2014). Probiotics have been suggested to work through various mechanisms in the gastrointestinal tract (Schmitz, 2021). Some of the proposed actions were associated with the inhibition of harmful bacteria growth in the gut by blocking their attachment to intestinal epithelial cells through mucus or mucin production and by competitive exclusion (Lee et al., 2003). Probiotics may also produce antimicrobial substances such as organic acids (Sanders et al., 2019) and stimulate the immune response of intestinal epithelial cells (Oelschlaeger, 2010). Currently, postbiotics, a new class of ‘biotics’ has been proposed. After years of multiple names and proposed definitions suggested, they have been recently defined according to ISAPP scientific discussion group as “preparation of inanimate microorganisms and/or their components that confers a health benefit on the host” (Salminen et al., 2021). Therefore, postbiotics include products of microbial metabolism, such as short-chain fatty acids (SCFA), enzymes, and others. They have been linked with enhancement of the intestinal barrier function and mucosal immunity, and anti-inflammatory properties (Cuevas-González et al., 2020). The mechanism of action is likely heterogeneous between individual postbiotics and has not been fully elucidated. However, similarly to probiotics, one discussed hypothesis for its role in modulating the immune system is through components of bacterial structures that are preserved in postbiotics. For example, it was observed that microbe-associated molecular patterns (MAMPs), such as peptidoglycans, can be recognized by pattern recognition receptors (PRRs) from the immune cells of the host generating several interactions that result in the inhibition of interleukin (IL) production; such as IL-6 and IL-8 (Teame et al., 2020).

The production of postbiotics requires the inactivation of microbial cells. This can be achieved by using several technological strategies, such as thermal treatment, irradiation, high pressure, ultra-violet rays, and sonication, which can alter the cell structure of the microbes promoting the inanimate state of the microorganism while preserving its functional properties (de Almada et al., 2016). Postbiotics present advantages compared to probiotics. Examples include potential lower infection risk once it may be a risk of cross contamination when probiotics are administered to vulnerable populations, other pharmacological advantages include facilitating transportation and storage, directly impacting shelf-life and further processing (Boyle et al.,2006; Piqué et al., 2019). In pet food formulating with postbiotics offers technological advantages over probiotics. The high temperatures used to control microbial contamination in pet food manufacturing are likely to impact the functionality of probiotics, if those are to be included before the extrusion or retorting processes. A postbiotic, originated from a heat-treated probiotic, would possibly provide the ease of incorporating this ingredient before processing as well as present a prolonged shelf-life stability (Yassine et al., 2021).

The use of postbiotics has been reported in vitro, and in vivo in mice, rats, pigs, chickens, and humans although very little information is currently available regarding the effects of pro- and postbiotics in feline nutrition and their effects on host metabolism. Thus, the objective of this research was to evaluate the longitudinal effects of daily supplementation of a probiotic and a heat-treated probiotic (herein referred as postbiotic) on biometrical measurements, fecal fermentative end-product concentrations, fecal microbiota, serum oxidative stress and inflammatory markers, and white blood cell gene expression of adult cats. It was hypothesized that the postbiotic supplementation would have comparable local (gut) and systemic effects to the probiotic supplementation, without detrimental outcomes to overall animal health.

## Materials and Methods

All animal care procedures were approved by the Kennelwood Inc. Animal Care and Use Committee. All methods were performed in accordance with the United States Public Health Service Policy on Humane Care and Use of Laboratory Animals.

### Study design and animals

Thirty-six adult domestic cats, characterized by an average body condition indicative of overweight status, [mean age = 4.98 ± 1.97 yr.; mean body weight (BW) = 5.30 ± 1.17 Kg; mean body condition score (BCS) = 6.33 ± 1.08, in a 9 point scale] participated in a longitudinal study using a double-blinded parallel randomized design. The experimental period was 90 d in length, preceded by a 60 d adaptation phase in which cats were fed the control diet alone. During the adaptation period dietary intake was adjusted to maintain bodyweight and cats were checked for overall health status. At this period, blood samples were collected to evaluate biomarkers of oxidative stress. These variables were then used to allocate cats into treatment according to their BW, BCS, age, and baseline serum oxidative stress biomarkers (Supplementary Tables S1 and S2). Two cats from the POST group were removed from the study after baseline due to unrelated medical conditions.

Throughout the study, sample collection occurred every 30 d, starting on day −30 (30 d before the implementation of treatments), day 0 (first day of treatment implementation), and then on days 30, 60, and 90. Animals were weighed and had their BCS and waist circumference (WC, cm) assessed weekly. Cats were group-housed for most of the day and had access to behavioral enrichments such as scratching posts and daily social interactions with care givers and research staff. They were individually fed for 1 hour in stainless steel cages twice a day at 0800 to 0900 and 1600 to 1700, and individually housed overnight, 1800 to 0800, with free access to water at all times.

### Treatments

Animals were fed a standard commercial complete and balanced extruded diet for adult cats (Cat Chow Complete Cat Food, Nestlé Purina PetCare Company, St Louis, USA) (Supplementary Table S3). Three interventions were tested with 12 animals distributed per group. Each intervention was delivered in 8 g of a standard commercial wet cat food (Friskies Paté Poultry Platter Wet Cat Food, Nestlé Purina PetCare Company, St Louis, USA) before the morning feed to ensure consumption of the full dose of either placebo (maltodextrin), probiotic, or postbiotic (ADM Biopolis S.L., Valencia, Spain). Subsequently, half of the daily ration of the dry cat food was provided to the cats during the morning feeding period. Food refusals were collected and recorded after each feeding period. The daily amount of food offered was calculated according to the NRC daily metabolizable energy requirements recommendations for adult cats, using the formula 100 kcal x kg BW^0.67^ for cats to maintain their current BW during the experimental period.

The experimental treatments tested were:

1. Control (CON): Extruded diet + Placebo (maltodextrin carrier)
2. Probiotic (PRO): Extruded Diet + probiotic source: *Bifidobacterium animalis* subsp. *lactis* CECT 8145 [daily dosage: 10^10^ CFU; however, plate counts conducted at the end of the study revealed that overtime dosage decreased to 6x10^9^. Original dose was based on previous human (Pedret et al., 2019) and rats (Carreras et al., 2018) studies].
3. Postbiotic (POST): Extruded Diet + Postbiotic (Postbiotic source: Heat-treated *Bifidobacterium animalis* subsp. *lactis* CECT 8145 daily dosage 10^10^ heat-treated cells)

### Sample collection

On each collection day of the experimental period, cats were individually housed with access to litter-free collection boxes. A fresh fecal sample was collected from each cat and processed within 15 minutes of defecation. Samples were weighed and their pH was measured. Aliquots of the samples were taken for measurement of phenols and indoles, SCFA, branched-chain fatty acids (BCFA), ammonia, and microbial analysis. Aliquots for fecal metabolite analysis were stored at −20°C. Aliquots for fecal microbiota analysis were collected on days 0 and 90 and placed in collection tubes prepared by ADM Biopolis SL (Paterna, Valencia, Spain) containing a stabilizing buffer (REAL stock solution, Durviz SL, Paterna, Valencia, Spain). Fecal samples were shaken for homogenization and stored at −20°C until further analysis.

A fasted blood sample was collected every 30 d throughout the experimental period. Cats were sedated with an intramuscular injection of 0.08 mL/kg of dexmedetomidine hydrochloride (0.5 mg/mL) (Dexdomitor, Orion corp., Espoo, Finland). Fifteen milliliters of blood were collected by jugular venipuncture. One milliliter of blood was aliquoted for complete blood cell count (CBC) analysis, 9 mL were aliquoted for serum chemistry, oxidative and inflammatory biomarker, and fructosamine analyses, and 5 mL were collected in PaxGene tubes (PreAnalytix, QIAGEN, Inc., Germantown, MD, USA) for RNA isolation. After blood collection, sedation was reversed by intramuscular injection of atipamezole hydrochloride (Antisedan, Orion Corp, Espoo, Finland) in a volume equivalent to the administered dexmedetomidine. CBC, fructosamine, and serum chemistry analyses were completed by the Clinical Pathology staff at the University of Illinois—College of Veterinary Medicine (Urbana, IL).

### Fecal fermentative end-products

Fecal SCFA and BCFA concentrations were determined using gas chromatography according to a modified method of Sunvold et al. (1995). A Hewlett-Packard (HewlettPackard, Avondale, PA) Model 5890A gas chromatograph equipped with a flame ionization detector on a column (1.8 m x 4 mm i.d.) packed with GP 10% SP-1200/1% H_3_PO_4_ on 80/100 chromosorb W AW (Supelco, Bellefonte, PA) was used to evaluate the diluted fecal samples for SCFA concentration using nitrogen at a flow rate of 45 mL/min as the carrier gas. The temperatures were 125, 175, and 180 °C for the oven, injection port, and detector port, respectively. Gas chromatography was also used to determine the fecal phenol and indole concentrations following the modified method of Flickinger et al. (2003). A Thermo Scientific TRACE 1300 Gas Chromatograph coupled with Flame Ionization Detector was used for this analysis, and a 1 µl sample was injected at 220 °C at splitless mode. The phenolic compounds were separated using a Nukol Supelco column (60 m length, 0.32 mm diameter) with a film thickness of 0.25 µm. For 1 minute the oven temperature was held at 150 °C, and then increased at 25 °C per min to 200 °C. The temperature was then held constant for 35 min. 5-methylindole was used as the internal standard, and samples were analyzed in duplicate. Ammonia concentration was evaluated using the method of Chaney and Marbach (1962).

### Fecal microbiome and bioinformatics

Total DNA extraction from fresh fecal samples was completed using a Mo-Bio PowerSoil kit (MO BIO Laboratories, Inc., Carlsbad, CA). A Qubit 3.0 Fluorometer (Life technologies, Grand Island, NY) was used to quantify DNA concentration prior to amplification and sequencing. Whole genome sequencing of the fecal microbiome was conducted by ADM Biopolis SL (Valencia, Spain). The Nextera XT sequencing libraries were prepared using the Illumina protocols. Once they were prepared an equimolar mixture was performed for sequencing in a NovaSeq 6000 of Illumina with the following configuration: 150x2 ‘Paired End’. The sequences were obtained from the sequencing platform using the software bcl2fastq v2.20.0.422 (Illumina) for ‘demultiplexing’. After the reads were filtered, presence of host genomes in the samples was filtered. For this, Genome assembly F.catus_Fca126_mat1.0 (Fca126) was used. Sequences with high homology to this genome were eliminated using the program NGLess v1.0.0-Linux64. The sequencing data supporting this study are openly available in the Sequence Read Archive (SRA) data under Submission No. PRJNA1222601 and reads statistics on the pre-processing steps were depict in the Supplementary Table S4.

The taxonomic assignment of the samples was obtained with the Metaphlan v2.0 program (Segata et al., 2012). The readings of each sample were aligned against single‐copy genetic markers present in almost all bacteria. From these alignments, the estimated number of reads contributed by a given clade for each identified taxa were obtained computationally. Metagenome was assembled from quality filtered reads from all samples using Spades (Bankevich et al., 2012). Contigs larger than 500 bp were used to predict genes using Prodigal (v2.6.3) (Hyatt et al., 2010). CAZY enzymes were annotated using dbCAN2 (Zhang et al., 2018).

### Serum leptin, oxidative stress, and inflammation biomarkers

Blood serum was used to determine the concentration of the oxidative stress biomarkers superoxide dismutase (SOD) and malondialdehyde (MDA) (measure by commercial bioassay, MyBioSource, San Diego, CA), the hormone leptin [enzyme-linked immunosorbent assay (ELISA) kit, MyBioSource, San Diego, CA], and 19 cytokines and chemokines (Milliplex Map Feline Cytokine/Chemokine Magnetic bead panel, Millipore, Billerica, MA). Cytokines evaluated were: FAS (pg/mL) = Cell surface death receptor; Flt-3L (pg/mL) = Fms-related tyrosine kinase 3 ligand; GM-CSF (pg/mL) = Granulocyte macrophage colony stimulating factor; IFN-gamma (pg/mL) = Interferon gamma; IL-1beta (pg/mL) = Interleukin-1 beta; IL-2 (pg/mL) = Interleukin-2; IL-4 (pg/mL) = Interleukin 4; IL-6 (pg/mL) = Interleukin 6; IL-8 (pg/mL) = Interleukin 8; IL-12(p40) (pg/mL) = Interleukin-12 (p40) subunit; IL-13 (pg/mL) = Interleukin 13; IL-18 (pg/mL) = Interleukin 18; KC (pg/mL) = Keratinocyte chemoattractant; MCP-1 (pg/mL) = Monocyte chemoattractant protein-1; PDGF-BB (pg/mL) = Platelet-derived growth factor-BB; RANTES (pg/mL) = regulated on activation, normal T cell expressed and secreted; SCF (pg/mL) = Stem cell factor; SDF-1 (pg/mL) = Stromal cell-derived factor 1; TNF-alpha (pg/mL) = tumor necrosis factor alpha.

### White blood cell gene expression

RNA from blood cells were isolated using PAXgene blood RNA Kit spectrophotometer (Nanodrop Te2chnologies, Wilmington, DE, USA). Using SuperScript IV, cDNA was synthesized by reverse transcriptase (Invitrogen, Carlsbad, CA, USA). DNA oligonucleotides of 25 nM were manufactured by IDT (Integrated DNA technologies Coralville, IA, USA) and used to from 33 selected primers (Supplementary Table S5) Primers were selected due to their importance in feline energy and lipid metabolism. Gene expression was quantified using the Fluidigm qPCR Biomark HD high throughput amplification system 48.48 qPCR amplification (Fluidigm Corporation, San Francisco, CA, USA) and EVA Control fluorescent dye (Roy J. carver Biotechnology Center; University of Illinois at UIUC). The quantification of relative mRNA abundance was determined by comparing results to the house-keeping gene RPL17 (ribosomal protein L17) to generate a Δct, and each animal on day 0 was used as their own control to calculate the ΔΔct, fold change values was generated by the $2^{-(\Delta \Delta ct)}$ formula, for each gene.

### Statistical analysis

Except for fecal microbiota, data were analyzed using the MIXED Model procedures of SAS version 9.4 (SAS Institute Inc., Cary, NC). Animal was used as the random effect, and treatment was used as the fixed effect in the statistical model. Day was used as the repeated measurement when appropriate. Data normality was checked using the UNIVARIATE procedure. Differences among treatments were assessed using Least Squares Means with a Tukey adjustment to account for type-1 experiment-wise error. The significance level was set at a probability of *P* < 0.05, and tendencies at 0.05 ≥*P* < 0.1 Pooled standard errors of the mean were obtained using the MIXED model procedure.

Alpha diversity indexes and richness were analyzed at species level using the Vegan packages from R (Oksanen et al., 2022). Beta diversity was performed with a PCoA clustering method and PERMANOVA based on a Bray-Curtis distances dissimilarity matrix using also Vegan R package. Abundance comparisons on taxonomy and CAZy enzymes were carried out with the R limma voom package (3.48.3v (Law et al., 2014)). P-values were adjusted based on the false discovery rate (FDR) of Benjamini and Hochberg. Heatmaps were constructed using ComplexHeatmap R package (Gu et al., 2016).

## Results

### Adaptation phase

Daily food intake, and weekly BW, BCS, and WC, as well as fecal scores during diet adaptation did not differ (*P* > 0.05) among treatments (Supplementary Table S1). Similarly, serum concentrations of leptin, MDA, and SOD (Supplementary Table S2), serum chemistry (Supplementary Table S6), and CBC (Supplementary Table S7) were not different (*P* > 0.05) among treatments. Among the 19 serum cytokines and chemokines analyzed during the adaptation phase (Supplementary Table S8), there were also no differences (*P* > 0.05) observed among treatments.

### Test phase

#### Food intake and biometric measurements during test period

Longitudinal assessment of food intake, BW, BCS and WC revealed no differences (*P* > 0.05) among dietary treatments (Table 1).

**Table 1.** Longitudinal assessment of biometrical measures and food intake of cats supplemented with probiotic and postbiotic

	Treatments													*P*-value		
	CON				POST				PRO							
**Items/Day**	**0**	**30**	**60**	**90**	**0**	**30**	**60**	**90**	**0**	**30**	**60**	**90**	**SEM^1^**	**Trt**	**Day**	Trt*Day
**BW (kg)^2^**	5.2	5.1	5.1	5.1	5.4	5.4	5.4	5.4	5.1	5.0	5.1	5.1	0.39	0.8329	0.2309	0.9508
**BCS^3^**	6.6	6.5	6.5	6.5	6.6	6.5	6.7	6.7	6.7	6.5	6.8	6.7	0.78	0.9535	0.0230	0.2776
**WC (cm)^4^**	34.5	33.2	31.9	29.3	33.1	33.4	30.9	30.5	34.2	33.5	32.0	29.9	1.73	0.9828	0.0001	0.3416
**FI (g/day)^5^**	59.7	59.9	61.3	64.6	65.5	63.0	65.6	66.3	57.9	57.6	60.9	62.8	5.20	0.7524	0.0168	0.9031
**Fecal score**	2.5	2.7	2.7	2.6	2.7	2.7	2.7	2.7	2.7	2.7	2.8	2.8	1.02	0.8088	0.2667	0.6963

1 SEM = Standard error of the mean

2 BW (kg) = Body weight;

3 BCS = Body condition score;

4 WC (cm) = Waist circumference;

5 FI (g/day) = Food intake.

#### Fecal fermentative end-products and fecal score during test period

**Figure 1.**
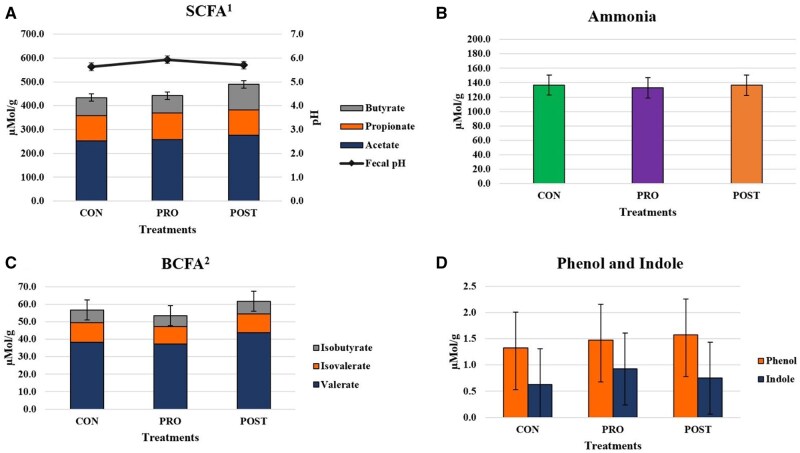
Average of fecal fermentative end-product concentrations of adult cats fed CON, PRO, and POST treatments. No significant difference time by treatment (*P *> 0.05) was observed. ^1^SCFA = Short chain fatty acids; ^2^BCFA = Branched chain fatty acids.

Fecal scores and pH did not differ (*P* > 0.05) among cats fed different treatments (Table 1 and Figure 1A). Fecal butyrate concentration (μmol/g, DMB) was greater (*P* < 0.05) in cats receiving the POST treatment (111.5 μmol/g) in comparison with the PRO treatment (77.3 μmol/g) for main treatment effect, although this difference was not observed by treatment over time, which indicates that the difference shown should not be interpreted as a result of the supplementation herein evaluated, once the different concentration was present at time zero and shown no statistical relevance when evaluated through difference from baseline (*P* > 0.05). There was no significant interaction of treatment by day, or main effect (*P* > 0.05) of treatment or day for fecal ammonia, acetate, propionate, BCFA, or phenol and indole concentrations (Figure 1B-D).

#### Fecal microbiota analysis during test period

##### Taxonomy

**Figure 2.**
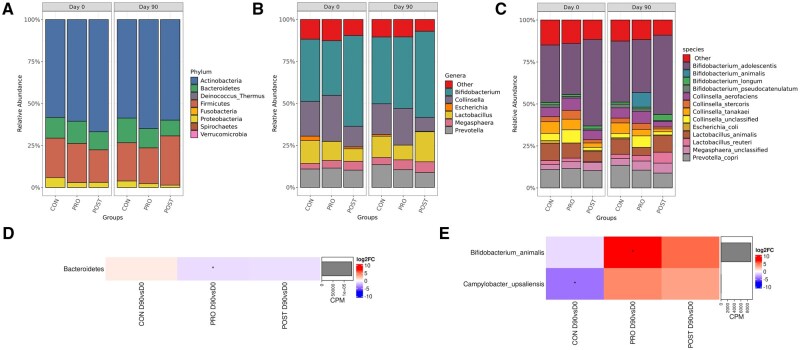
Fecal microbiota relative abundance of the taxonomical profile at a phylum (A), genera (B), and species (C) level between days 0 and 90 of adult cats fed CON, PRO, and POST treatments. Heatmap of the log2 fold change of fecal microbiota taxa abundance at the phylum (D), species (E) level between days 0 and 90 of adult cats fed CON, PRO, and POST treatments. Taxa abundance are shown increased (red color) or decreased (blue color) in each comparison. The (*) indicates comparisons with *P*-value < 0.05. CPM values for each species are indicated as barplots on the right.

A total of 106 species were observed in fecal samples analyzed. The most abundant phyla obtained were Actinobacteria (61.59 ± 22.54%), Firmicutes (23.13 ± 17.77%), Bacteroidetes (12.04 ± 8.96%), and Proteobacteria (3.25 ± 3.92%). Overall, the most abundant genera were *Bifidobacterium* (42.66 ± 28.90%), *Collinsella* (18.42 ± 16.11%), *Lactobacillus* (11.80 ± 17.22%), and *Prevotella* (10.97 ± 8.26%). At species level the most abundant were *Bifidobacterium adolescentis* (38.31 ± 28.88%), *Segatella copri* (10.97 ± 8.26%), and *Ligilactobacillus animalis* (formerly *Lactobacillus animalis*) (8.19 ± 12.47%) (Figure 2).

At phylum level there was a decreased (*P* < 0.05) relative abundance of Bacteroidetes in the PRO (−1.82) group and a tendency (*P* < 0.10) of increased relative abundance in the CON group over time (Figure 2). At the family level, *Campylobacteraceae* abundance significantly decreased (*P* < 0.05) on CON group over time. No significant (*P* > 0.05) time dependent changes were obtained at genus level. At species level, the relative abundance of *Bifidobacterium animalis* and *Campylobacter upsaliensis* varied significantly with time; *B. animalis* increased (*P* < 0.05) over time in the PRO group and decreased in CON group, whilst *C. upsaliensis* decreased (*P* < 0.05) in the CON group only (Figure 2).

##### Alpha and beta diversities.

**Figure 3.**
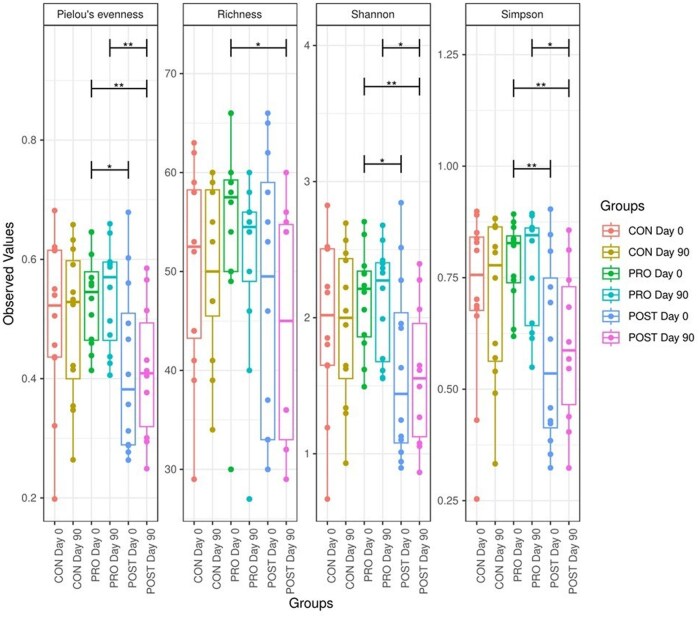
Boxplots of the richness and alpha diversity index values according to the groups of cats fed CON, PRO, POST treatments on days 0 and 90. The differences between groups were tested according to pairwise ANOVA tests. With significant differences indicated by asterisks (*P-value  < 0.05; ** *P*-value  < 0.01).

Alpha diversity values were lower (*P* < 0.05) in POST group treatment compared to PRO, for Simpson, Shannon, and Pielou’s evenness index. Alpha diversity of the CON group did not differ (*P* > 0.05) from PRO or POST groups (Figure 3).

The individual cats as a variable explained 87% of the total variability of permutational multivariate analysis of variance (PERMANOVA), there was no significant difference or clear clustering overserved in this beta diversity for the 3 treatments.

### Carbohydrate-active enzymes (CAZy) genes

**Figure 4.**
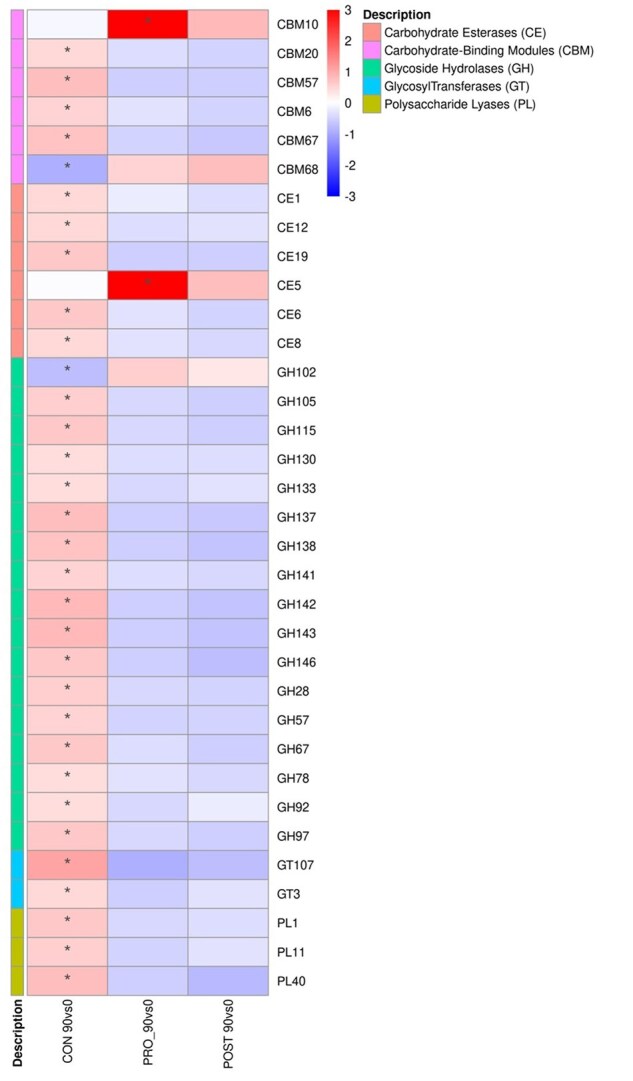
Heatmap of the ‘log2 fold change’ of taxa abundance at the CAZy functional level of adult cats fed CON, PRO, and POST treatments, from day 0 to 90. CAZy categories are shown increased (red color) or decreased (blue color) for CAZy genes. The (*) indicates comparisons with *P*-value < 0.05.

**Figure 5.**
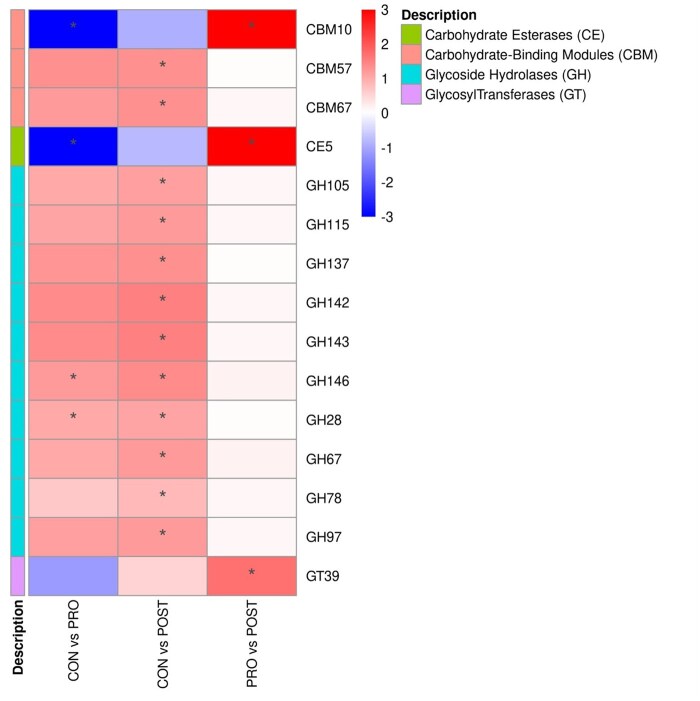
Heatmap of the ‘log2 fold change’ of taxa abundance at the CAZy functional level of adult cats fed CON, PRO, and POST, between treatments. CAZy categories are shown increased (red color) or decreased (blue color) for CAZy genes. The (*) indicates comparisons with *P*-value < 0.05.

An increase (*P* < 0.05) of several CAZy families over time (day 0 vs. 90) was observed in the CON group, in pathways related to carbohydrate-binding molecules (CBM) 5 families, carbohydrate esterases (CE) 5 families, glycoside hydrolases (GH) 17 families, glycosyl tranferases (GT) 2 families, and polysaccharide lyases (PL) 3 families. Whilst the PRO and POST groups showed a tendency of abundance decrease over time (*P* > 0.05). An increase (*P* < 0.05) was observed for cats fed the PRO only for the genes CBM10 (The cellulose-binding function) of the CBM family, and CE5 (acetyl xylan esterase; cutinase) carbohydrate esterases family, when compared with CON treatment (Figures 4 and 5).

### Serum leptin, oxidative stress, and inflammatory biomarkers

Longitudinal analysis of serum leptin, MDA, and SOD concentrations revealed no treatment by time interaction or main treatment effect (*P* > 0.05) of cats being fed different dietary treatments (Supplementary Table S9). Among the 19 serum cytokines and chemokines analyzed, no treatment by time interaction was observed (*P* > 0.05), and only a few measures differed among treatments (Table 2). Cats on CON treatment (9.2 pg/mL) had a lower (*P* < 0.05) GM-CSF concentration than cats on the POST treatment (23.3 pg/mL). In contrast, serum IL-12(p40) concentration was higher (*P* < 0.05) on treatment CON (658 pg/mL) than of cats on the POST treatment (432 pg/mL). Serum RANTES concentration was greater (*P* < 0.05) in cats on the CON (82.5 pg/mL) treatment in contrast with cats on the POST (53.6 pg/mL) and PRO (53.6 pg/mL) treatments. Serum Flt-3L concentration, however, tended to be higher (*P* = 0.0546) on CON treatment (111 pg/mL) in comparison with POST (77 pg/mL). In contrast, IL-13 (*P* = 0.0763), tended to be lower for CON treatment (19.1 pg/mL) compared to POST (28.4 pg/ML). The 5 analytes were evaluated for difference of baseline, for days 30, 60, and 90, no statistical difference was observed (*P* > 0.05), which indicates that the difference shown should not be interpreted as a result of the supplementation herein evaluated, once the different concentration was present at time zero.

**Table 2.** Longitudinal assessment of serum cytokines and chemokines concentrations of cats supplemented with probiotic and postbiotic

Day	Treatments															
	CON				POST				PRO				*P*-value			
	0	30	60	90	0	30	60	90	0	30	60	90	SEM^1^	Trt	Day	Trt*Day
***Analytes, pg/mL***																
**FAS^2^**	20.4	14.4	19.9	18.7	26.5	23.3	19.7	14.1	14.6	21.9	15.6	22.1	6.46	0.0549	0.9542	0.7260
**Flt-3L^3^**	117.8	100.3	119.2	107.9	71.0	66.5	78.4	92.2	82.5	86.5	92.3	89.5	20.11	0.0546	0.8646	0.9899
**GM-CSF^4^ ,^#^**	11.3	8.8	13.0	10.1	21.8	29.4	17.8	26.8	20.8	12.9	11.7	22.5	5.12	0.0027	0.6003	0.5682
**IFN**γγ **^5^**	283.3	294.4	281.9	287.8	287.6	273.6	270.1	332.7	415.8	378.1	462.3	471.7	155.29	0.5233	0.9041	0.9974
**IL-1**ββ **^6^**	45.9	48.9	44.3	43.0	40.5	34.8	33.7	51.8	53.3	57.7	73.4	61.0	19.21	0.7844	0.8167	0.8878
**IL-2^7^**	30.8	33.1	31.6	33.0	43.3	45.2	36.0	43.5	40.8	27.9	34.1	45.4	10.48	0.5049	0.9106	0.9349
**IL-4^8^**	640.2	637.6	609.8	640.3	540.4	541.6	478.0	616.1	1070.1	867.5	1005.1	1111.8	340.23	0.3440	0.9726	0.9911
**IL-6^9^**	330.6	289.9	303.4	386.1	240.5	226.1	208.5	273.1	356.9	313.0	476.0	492.9	146.19	0.9018	0.5794	0.9749
**IL-8^10^**	53.6	48.6	58.2	56.1	39.8	40.2	42.6	50.7	52.9	46.4	55.2	55.4	11.85	0.4678	0.7026	0.9833
**IL-12(p40)^11^ ,^#^**	688.2	640.8	685.2	623.5	390.5	343.9	510.4	499.6	456.7	578.4	521.4	766.2	156.94	0.0392	0.9247	0.9785
**IL-13^12^**	19.8	18.4	19.5	18.8	29.4	27.0	27.8	29.2	21.7	22.1	21.7	25.6	5.64	0.0763	0.9907	0.9962
**IL-18^13^**	144.9	148.5	150.6	123.4	224.7	208.6	188.9	265.1	291.8	149.8	260.8	258.5	69.11	0.1418	0.7823	0.9261
**KC^14^**	30.6	24.8	26.5	17.1	18.8	20.2	34.2	39.8	32.8	35.4	30.7	38.9	14.14	0.6271	0.9894	0.8717
**MCP-1^15^**	1638.3	1563.9	1863.7	1538.0	1704.8	1741.5	1715.3	1921.4	1851.4	1571.0	1893.9	2044.6	308.18	0.6801	0.8338	0.9549
**PDGF-BB^16^**	2158.2	1780.4	2447.4	2238.9	1504.9	1720.4	1794.5	2446.7	1660.2	1163.8	1863.5	1885.5	444.96	0.0694	0.2731	0.9062
RANTES^17^ ,^#^ ,^*^	76.9	77.2	92.2	83.5	42.1	44.9	51.9	53.7	56.3	50.2	51.9	56.0	12.60	0.0003	0.8243	0.9907
**SCF^18^**	255.2	221.6	226.6	225.0	172.2	171.2	181.4	190.0	218.7	238.3	269.5	226.3	65.27	0.5438	0.9387	0.9930
**SDF-1^19^**	4335.2	2909.2	3101.9	3965.9	2746.4	2754.5	3686.6	2908.1	2467.1	3585.7	3075.8	3159.5	656.23	0.4174	0.9647	0.4374
**TNF** **^20^**	349.8	246.4	262.0	346.0	166.6	137.9	177.6	186.8	369.9	327.5	419.3	320.0	144.66	0.2305	0.9324	0.9879

1 SEM = Standard error of the mean

2 FAS = Cell surface death receptor;

3 Flt-3L = Fms-related tyrosine kinase 3 ligand;

4 GM-CSF = Granulocyte macrophage colony stimulating factor;

5 IFNγ = Interferon γ;

6 IL-1β = Interleukin-1 beta;

7 IL-2 = Interleukin-2;

8 IL-4 = Interleukin 4;

9 IL-6 = Interleukin 6;

10 IL-8 = Interleukin 8;

11 IL-12(p40 = Interleukin-12 (p40)subunit;

12 IL-13 = Interleukin 13;

13 IL-18 = Interleukin 18;

14 KC = Keratinocyte chemoattractant;

15 MCP-1 = Monocyte chemoattractant protein-1;

16 PDGF-BB = Platelet-derived growth factor-BB;

17 RANTES = regulated on activation, normal T cell expressed and secreted;

18 SCF = Stem cell factor;

19 SDF-1 = Stromal cell-derived factor 1;

20 TNFα = tumor necrosis factor alpha.

# Treatments CON and POST were significantly different (*P* < 0.05).

* Treatments CON and PRO were significantly different (*P* < 0.05).

### White blood cell gene expression during test period

Longitudinal analysis of white blood cell gene expression revealed no treatment by day interaction (*P* > 0.05) or main treatment effect differences for any of the genes analyzed.(Supplementary Tables S10 and S11).

## Discussion

### Baseline measurements

There were no remarkable differences observed during baseline among treatments. These results were expected, as cats were allocated to different treatments based on age, biometrical measurements, and oxidative stress biomarkers, which was determined during the adaptation phase to minimize the impact of individual variation on variables of interest in this study.

### Biometrical measurements and food intake

Supplementation of *Bifidobacterium animalis* subsp*. lactis* CECT 8145 in a trial with obese humans promoted BW and WC reduction (Pedret et al., 2019). Similarly, the use of *Bifidobacterium animalis* subsp*. lactis* CECT 8145 in *Caenorhabditis elegans* demonstrated potential fat-reducing properties linked to the insulin like growth factor-1 (IGF-1) pathway (Balaguer et al., 2022). In contrast, previous study in cats testing *Enterococus faecium* SF68 did not lead to BW loss or reduction in daily food intake during probiotic consumption (Kathrani et al., 2016). Zini et al. (2021) proposed that BW gain and loss in adult cats does not affect IGF-1 concentrations in the body, which may, at least in part, explain the divergent findings in the literature. Studies utilizing probiotics to promote weight loss have been successful in humans and mice, but no study with cats has yet shown the same response. Experimental design, time of exposure, dosage, and probiotic strain and form utilized could also explain discrepancies reported.

### Fecal fermentative end-products

The concentrations of butyrate observed (44.5–123.3 μMol/g) in the present study were numerically higher than those reported in a study using a multi-strain yeast probiotic with 12 cats (n = 6 per group), in which cats were fed approximately 2.0 × 10^10^ CFU/g *Saccharomyces boulardii* and approximately 2.5 × 10^10^ CFU/g of *Pediococcus acidilactici* (4-6 µMol/g), cats were fed control diet and multistrain supplemented diet for 28 d (Li et al., 2023). However, fecal butyrate concentrations were similar to values reported by another study in which cats were fed 3 different extruded diets supplemented with either resistant starch, or a fiber-prebiotic-probiotic blend-containing formula, or a fiber-prebiotic-probiotic blend + immuno-modulating ingredient-containing formula, for 28 d, (60.3–212.2 µMol/g) (Lee et al., 2022). Colonocytes will, preferentially, use butyrate as an energy source, which stimulates the growth and development of gut epithelial cells, helping maintain the structure, integrity, and function of the gut lining (Wong et al., 2006; Liu et al., 2021). Butyrate can also suppress the transcription and differentiation of various immune cells, by inhibiting histone deacetylase. This anti-inflammatory effect is crucial in preserving the balance between the immune response to pathogenic bacteria in the gut and tolerance of commensal bacterial species (Tizard and Jones, 2018; Dalile et al., 2019; Siddiqui and Cresci, 2021). The fecal butyrate concentration of POST group did not differ from the control group, although not statistically significant all treatments had fecal butyrate concentration numerically decrease at day 60 with subsequent numerically increase for both PRO and POST treatment groups on day 90, whilst the CON group remained numerically lower.

### Fecal microbiota

In the present study, the most abundant phyla observed were Actinobacteria, Firmicutes, Bacteroidetes, and Proteobacteria. This is consistent with previous publication by Deng and Swanson (2015), which also identified these phyla as being dominant in the healthy feline gut. Although Actinobacteria has been reported an one of the 4 main phyla in the feline microbiome, other studies have reported lower abundance of Actinobacteria and higher abundance of the Firmicutes, Bacteroidetes, and Proteobacteria phyla (Cockburn and Koropatkin, 2016). Actinobacteria phylum organisms play important roles in nutrient metabolism and immune function, an example of a common genus of Actinobacteria in the feline gut is *Bifidobacterium*, which has been associated with health benefits such as improved immune function (Fukuda et al., 2011; Cheng et al., 2021), The GI microbiome composition can be affected by several factors, such as diet, age, and health status. The phyla Actinobacteria and the genus *Bifidobacterium* presented higher abundance in the present study compared to other published reports. Although more research is needed to fully understand the exact explanation for contrast, possible causes may be associated to the specific population of cats utilized in this study and the methods for fecal microbiota data analysis.

The abundance of Bacteroidetes phylum was lower in the PRO group when compared to the CON group. A reduction in Bacteroidetes abundance could be associated with gut disease, particularly diarrhea (Suchodolski et al., 2015). However, in the present study, fecal samples from animals were regularly scored and no differences were observed in their quality. At the species level, cats fed the PRO supplementation showed an increase in the abundance of *Bifidobacterium animalis*, whereas the CON group had a decreased abundance of this taxon. Although the fecal microbiota analysis did not evaluate specifically strain CECT 8145, this result suggests that the supplementation of the live *Bifidobacterium animalis* subsp *lactis* CECT 8145 may have contributed to the increased abundance of that species, potentially surviving through the feline gastrointestinal tract. Cats with inflammatory bowel disease (IBD) have altered gut microbiome composition compared with healthy cats. It was reported that Bifidobacterium spp. and Bacteroides spp counts, considered beneficial microbes, were lower in cats with IBD (Janeczko et al., 2008; Marsilio et al., 2019). A study conducted by O’Mahony et al. (2009) reported that daily supplementation in dogs of *Bifidobacterium animalis* strain AHC7 for 6 weeks promotes microbiome modulation and enhancement of gastrointestinal health by reducing the total clostridia levels and *Clostridium difficile* numbers of fecal samples.

Relative abundance of *Campylobacter upsaliensis*, a bacterium that has been linked to gut disease in humans, decreased in fecal samples of cats in the CON group. This species has been linked to potentially gut disease in cats, and clinical symptoms were mainly observed in young animals, those from intensive housing backgrounds, and animals with concurrent diseases (Hald and Madsen, 1997). In the present study, the presence and relative abundance of this bacterium did not lead to health problems.

Although no differences were observed on alpha and beta diversities, functional changes were detected in specific genes. CAZy genes indicated a few significant differences. The CAZy database describes the families of structurally related catalytic and carbohydrate-binding modules (or functional domains) of enzymes that degrade, modify, or create glycosidic bonds (Drula et al., 2022). Overall, cats supplemented with PRO and POST had similar response to each other, in contrast with the CON group. Families related to pathways such as CE, CBM, and GH, had the most differences associated to the CON group, increasing for all families but two, CBM68 and GH102 over time. Glycoside hydrolases, enzymes that catalyze the hydrolysis of the glycosidic linkage of glycosides (Marar and Garg, 2020), were the most prevalent family to increasing over time among the CAZy families in the CON fed group. In gut microbes, GHs have crucial roles in breaking down complex carbohydrates and processing various exogenous and endogenous glycoconjugates (Pellock et al., 2018). Whilst for the PRO fed cats, CBM10 abundance was increased over time, carbohydrate-binding modules are non-catalytic proteins with the capacity to bind to soluble and crystalline carbohydrate. In this context, CBM10 plays a crucial role in enhancing the activity of cellulases and lytic polysaccharide monooxygenases against crystalline cellulose. It has been suggested that this ability to enhance catalytic function is achieved by increasing the local concentration or proximity of the enzyme to the substrate (Gill et al., 1999; Crouch et al., 2016).

### Serum leptin, oxidative stress, and inflammatory biomarkers

Cytokines such as IL-6, TNF-alpha and IL-1beta, known to be important cytokines in a pro-inflammatory status (Safi et al., 2017), showed relatively low values, and were not different with time for any treatments, which indicate that animals fed the CON, PRO, and POST diets were not going through acute inflammation during the trial. Although there are currently no established reference values in the literature for these specific cytokines and chemokines as markers of a healthy status in adult cats.

## Conclusion

This study showed that probiotic and postbiotic *Bifidobacterium animalis* subps *lactis* CECT 8145 had no adverse effect on feline health during the 90 d of supplementation and were well tolerated when orally administered. Cats fed PRO treatment had an increased relative abundance over time of *Bifidobacterium animalis* suggesting a possible successful colonization in the feline gut. Analysis of the CAZy genes in cats fed PRO and POST treatments showed similar response to each other as opposed to the CON group, indicating the potential for comparable functionality. Overall, the study indicates that the PRO and POST supplementation had similar response in oral administration for adult cats. Considering these results the use of the selected probiotic and postbiotic might be beneficial to cats. Although more studies are required to support these claims and the effects of probiotics and postbiotics in feline nutrition, such as further characterization of the functional ingredient’s composition.

## Supplementary Material

skaf355_Supplementary_Data

## Abbreviations:

ACLY: ATP citrate lyase
ADIPOQ: adiponectin, C1Q and collagen domain containing
ADIPOR1: adiponectin receptor 1
ADM: Archer Daniels Midland Company
ALT: alanine transaminase
BCFA: branched-chain fatty acids
BCS: body condition score
BW: body weight
CBC: blood cell count
CCR2: C-C motif chemokine receptor 2
cDNA: complementary deoxyribonucleic acid
CH2: major allergen I, polypeptide chain 2
CPK: creatine phosphokinase
DMB: dry matter basis
DNA: deoxyribonucleic acid
ELISA: enzyme linked immunosorbent assay
FAS: cell surface death receptor
FASN: fatty acid synthase
FDR: false discovery rate
FFAR4: G protein coupled receptor 120/free fatty acid receptor 4
Flt-3L: Fms-related tyrosine kinase 3 ligand
GM-CSF: granulocyte macrophage colony stimulating factor
GPR: G-protein-coupled receptors
HCAR2: hydroxycarboxylic acid receptor 2
HPRT: hypoxanthine guanine phosphoribosyl transferase
IFNγ: interferon gamma
ILCs: innate lymphocyte cells
IL-12(p40): interleukin-12 (p40) subunit
IL-13: interleukin 13
IL-18: interleukin 18
IL-1β: interleukin-1 beta
IL-2: interleukin-2
IL2RA: interleukin 2 receptor subunit alpha
IL-4: interleukin 4
IL6: interleukin 6
IL-8: interleukin 8
INSR: insulin receptor
IRS1: insulin receptor substrate 1
IRS2: insulin receptor substrate 2
KC: keratinocyte chemoattractant
LEP: precursor leptin
LIPE: hormone sensitive lipase
LPL: lipoprotein lipase
MAMPs: microbe-associated molecular patterns
MCH: mean corpuscular hemoglobin
MCHC: mean corpuscular hemoglobin concentration
MCP-1: monocyte chemoattractant protein-1
MDA: malondialdehyde
MDH1: cytosolic malate dehydrogenase
MDH2: malate dehydrogenase 2 (mitochondrial)
mRNA: messenger ribonucleic acid
PDGF-BB: platelet-derived growth factor-BB
PIK3R1: phosphatidylinositol 3-kinase regulatory subunit alpha
PPARG: peroxisome proliferator activated receptor gamma 1
PRRs: pattern recognition receptors
qPCR: quantitative polymerase chain reaction
RANTES: regulated on activation, normal T cell expressed and secreted
RETN: resistin
RNA: ribonucleic acid
RPL17: ribosomal protein L17
SCF: stem cell factor
SCFA: short-chain fatty acids
SDF-1: stromal cell-derived factor 1
SERPINE1: plasminogen activator inhibitor 1
SLC2A1: solute carrier family 2 member 1 (GLUT 1)
SLC2A4: solute carrier family 2 member 4 (GLUT 4)
SOCS5: suppressor of cytokine signaling 5
SOD: superoxide dismutase
SREBF1: sterol regulatory binding protein-1c-like
TLR4: toll like receptor 4
TNFα: tumor necrosis factor alpha
WC: waist circumference
YWHAZ: tyrosine 3-monooxygenase/tryptophan 5-monooxygenase activation protein zeta polypeptide
