# Supplementary material for: Effects of supplementation with Bifidobacterium animalis subsp. lactis CECT 8145—in live probiotic and heat-treated postbiotic form—on fecal metabolites, fecal microbiota, blood metabolites and systemic biomarkers of oxidative stress and inflammation, and white blood cell gene expression of adult cats
Source: J Anim Sci. 2025 Oct 21;103:skaf355. doi: 10.1093/jas/skaf355 (PMC12664571; doi:10.1093/jas/skaf355)
Supplement: skaf355_Supplementary_Data [file skaf355_supplementary_data.zip › Supplemetary figures and tables_AL.docx]

**Suppl.Table 1.** Mean body weight, biometrical measures, food intake, and fecal scores of cats supplemented with probiotic and postbiotic during adaptation period (day -30).

|  | **Treatments** | | |  |  |
| --- | --- | --- | --- | --- | --- |
| **Items/Day** | **CON** | **POST** | **PRO** | **SEM^1^** | **p-value** |
|  |  |  |  | **Trt** | **Trt** |
| **BW (kg)^2^** | 5.2 | 5.5 | 5.1 | 0.36 | 0.7120 |
| **BCS^3^** | 6.4 | 6.3 | 6.3 | 0.36 | 0.9864 |
| **WC (cm)^4^** | 36.7 | 35.4 | 37.4 | 1.64 | 0.6700 |
| **FI (g/day)^5^** | 67.3 | 76.8 | 66.5 | 5.21 | 0.3091 |
| **Fecal score^6^** | 2.6 | 2.7 | 2.6 | 0.11 | 0.9852 |

^1^SEM = Standard error of the mean

^2^BW = Body weight; ^3^BCS = Body condition score; ^4^WC = Waist circumference; ^5^FI = Food intake; ^6^Fecal scores recorded during the first 7 days of diet adaptation period

**Suppl. Table 2.** Serum concentration of serum leptin and oxidative stress biomarkers of cats supplemented with probiotic and postbiotic during adaptation period (day -30).

|  | **Treatments** | | |  | **p-value** |
| --- | --- | --- | --- | --- | --- |
| **Analytes** | **CON** | **POST** | **PRO** | **SEM^1^** | **Trt** |
| **Leptin, ng/ml** | 0.6 | 0.6 | 0.5 | 0.06 | 0.6131 |
| **MDA^2^,** $\boldsymbol{\mu}$**Mol/L** | 24.8 | 26.1 | 24.6 | 1.94 | 0.8414 |
| **SOD^3^, ng/ml** | 0.3 | 0.2 | 0.2 | 0.05 | 0.2741 |

^1^SEM = Standard error of the mean.

^2^MDA ($\mu$Mol/L) = Malondialdehyde; ^3^SOD (ng/ml) = Superoxide dismutase.

**Suppl.Table 3.** Guaranteed analysis of commercial diets used in cats supplemented with probiotic and postbiotic.

| **Items, as is basis** | **Dry^1^** | **Wet^2^** |
| --- | --- | --- |
| **Crude Protein %** | 32.00 | 9.00 |
| **Crude Fat %** | 12.00 | 5.00 |
| **Crude Fiber %** | 3.00 | 1.00 |
| **Ash %** | ---- | 3.50 |
| **Moisture %** | 12.00 | 78.00 |
| **Taurine %** | 0.15 | 0.05 |
| **Metabolizable energy (ME);Kcal/g** | 3.08 | 1.16 |

^1^Dry diet: Cat Chow Complete Cat Food (Nestlé Purina PetCare Company, St. Louis, USA). INGREDIENTS: Chicken by-product meal, ground yellow corn, corn gluten meal, whole grain wheat, rice, soy flour, beef fat preserved with mixed-tocopherols, chicken, fish meal, liver flavor, phosphoric acid, calcium carbonate, salt, potassium chloride, choline chloride, MINERALS [zinc sulfate, ferrous sulfate, manganese sulfate, copper sulfate, calcium iodate, sodium selenite], taurine, VITAMINS [Vitamin E supplement, niacin (Vitamin B-3), Vitamin A supplement, calcium pantothenate (Vitamin B-5), thiamine mononitrate (Vitamin B-1), riboflavin supplement (Vitamin B-2), Vitamin B-12 supplement, pyridoxine hydrochloride (Vitamin B-6), folic acid (Vitamin B-9), Vitamin D-3 supplement, biotin (Vitamin B-7), menadione sodium bisulfite complex (Vitamin K)], DL-Methionine, Red 40, Yellow 5, Blue 2.

^2^Wet diet: (Friskies Paté Poultry Platter Wet Cat Food, Nestlé Purina PetCare Company, St. Louis, USA). INGREDIENTS: Poultry, water, meat by-products, liver, poultry by-products, fish, rice, artificial and natural flavors, MINERALS [potassium chloride, magnesium proteinate, zinc sulfate, ferrous sulfate, manganese sulfate, copper sulfate, potassium iodide], guar gum, carrageenan, choline chloride, taurine, VITAMINS [thiamine mononitrate (Vitamin B-1), Vitamin E supplement, niacin (Vitamin B-3), calcium pantothenate (Vitamin B-5), Vitamin A supplement, menadione sodium bisulfite complex (Vitamin K), pyridoxine hydrochloride (Vitamin B-6), riboflavin supplement (Vitamin B-2), Vitamin B-12 supplement, biotin (Vitamin B-7), folic acid (Vitamin B-9), Vitamin D-3 supplement], salt.

**Suppl.Table 5**. Details of selected primers of importance in feline metabolism

| **Gene** | **Accession number** | **Forward primer (5′ → 3′)** | **Reverse primer (5′ → 3′)** | **Tm (°C)** | **Frag. (bp)** |
| --- | --- | --- | --- | --- | --- |
| **HPRT^1^** | EF453697 | ACTGTAATGACCAGTCAACAGGGG | TGTATCCAACACTTCGAGGAGTCC | 60.0 | 210 |
| **RPL17^2^** | AY738264 | CTCTGGTCATTGAGCACATCC | TCAATGTGGCAGGGAGAGC | 58.0 | 108 |
| **YWHAZ^3^** | EF458621 | GAAGAGTCCTACAAAGACAGCACGC | AATTTTCCCCTCCTTCTCCTGC | 65.0 | 115 |
| **ACLY^4^** | GU332841.1 | TCAAGAAGGCAGACCAGAAGG | TACAGCTTGGAGGCCAGGATG | 62.0 | 127 |
| **ADIPOQ^5^** | NM_001085438.1 | CCGGGTGAAAAGGGTGAGAA | CCGACTCTCCAATCCCACAC | 60.0 | 182 |
| **ADIPOR1^6^** | NM_001134681.1 | TGCCAGTAACAGGGAAGCTG | ATTGTCCTTCAGCCAGTCGG | 60.0 | 274 |
| **FASN^7^** | AB436620.1 | TACTGGAGGGGCCAGTGCATCA | GTCCCGAGATGGTCACTGTGTC | 65.0 | 151 |
| **SLC2A1^8^** | XM_003989965.6 | ATTGTGGCTGAACTCTTCAG | CCAGGAGTACGGTGAAGATG | 57.4 | 157 |
| **SLC2A4^9^** | DQ640899 | GGCCATCGTCATTGGCATTC | CGGCTGATGTAGAGGTAGCG | 60.0 | 168 |
| **FFAR4^10^** | LC014926.1 | TGATGCCGGGATTGCTCATT | ACATGGCTCCCTTTTCTGGG | 60.0 | 387 |
| **HCAR2^11^** | NM_001309044.1 | ATCTGCCTGCCATTCTTGAC | CCACGGTGAGGAAGATGATG | 60.0 | 75 |
| **LIPE^12^** | AY999302 | TGTCCTTCGGGGAGCACTAT | TGGTGGATGTCATGTTCGCT | 60.0 | 213 |
| **INSR^13^** | DQ835565.1 | TTGTGGAGCGTTGTTGGACA | CAGGACACCGCACTTGGT | 60.0 | 152 |
| **IRS1^14^** | AB436530 97 | ACCTGCGTTCAAGGAGGTCTG | CGGTAGATGCCAATCAGGTTC | 62.0 | 81 |
| **IRS2^15^** | AB436531 | TGGCAGGTGAACCTGAAGC | GAAGAAGAAGCTGTCCGAGTGG | 61.0 | 177 |
| **IFNG^16^** | NM_001009873.1 | TGCAAGTA ATCCAGATGTAGCAG | GTTTTATC ACTCTCCTCTTTCCAG | 58.0 | 81 |
| **IL1B^17^** | NM_001077414.1 | TGGCCCCGAAAAGATGAAGG | TCATCCTGGAGGGGTTGTGA | 60.0 | 186 |
| **IL6^18^** | M_001009211.2 | GGCACTGGCAGAAAACAACC | CTTCTACGGTTGGGACAGGG | 61.0 | 269 |
| **LPL^19^** | U42725.1 | ACTTGCCACCTCATTCCTGG | CCGGTAGGCCTTACTAGGGT | 60.0 | 714 |
| **MDH1^20^** | AB113364 | GGTGCAGCCTTGGAGAAATATG | CAGTCAGGCAGTTGGTATTGG | 60.0 | 82 |
| **MDH2^21^** | NM_001278853.1 | AAAGTAGCCGTGTTAGGGGC | CCGTAATCGGGATGGTGGAG | 60.0 | 379 |
| **SERPINE1^22^** | DQ835567.1 | CAGAGCCAGGTTCATCGTCAA | AGTAGAGGGCATTCACCAGCAT | 61.0 | 122 |
| **PPARG^23^** | NM_001113176.1 | AAGAGCGGACCCGATGGTT | AATAAGGTGGGGATGCAGGC | 60.0 | 95 |
| **PIK3R1^24^** | AB436617 | GCATTAAACCAGACCTCATTCAGC | GCGAGTATTGGTCTTCAGTGTTCTC | 62.0 | 132 |
| **RETN^25^** | LC064405.1 | ATGAGGCCATCCACGAGAAG | GCTCAGGCCAAAGTTCCTTA | 59.0 | 77 |
| **SREBF1^26^** | GU332842.1 | CCATTGCCGCTGACTATCCT | CTGGGATAGCCCAACACAGG | 60.0 | 125 |

**Suppl.Table 5 (Cont.)**. Details of selected primers of importance in feline metabolism

| **Gene** | **Accession number** | **Forward primer (5′ → 3′)** | **Reverse primer (5′ → 3′)** | **Tm (°C)** | **Frag. (bp)** |
| --- | --- | --- | --- | --- | --- |
| **TNF^27^** | NM_001009835.1 | CACATGGC CTGCAACTAATC | AGCTTCGG GGTTTGCTACTAC | 60.00 | 104 |
| **TLR4^28^** | NM_001009223.1 | GCCTGAGGAGGAAACACTCC | AAACCCCATGAGGTCAGCAG | 60.00 | 161 |
| **SOCS5^29^** | XM_003983962.6 | CTACACGCCTCCGGATTGG | TATCGTGCACCGAGCTTACG | 60.25 | 568 |
| **IL2RA^30^** | NM_001009355.1 | GGGAATCCTCACATTCGTCGT | GAGGAGTGGCTCGAGTTTCC | 60.11 | 201 |
| **CCR2^31^** | NM_001097580.1 | GGCTCTATCACGTCGGCTAC | GAAGGGCGGCAGGAGTAAAA | 60.32 | 226 |
| **CH2^32^** | NM_001048154.1 | AAGATGGCGGAAACTTGCCC | CGTTCTCCACGTAGCAATCCT | 60.14 | 154 |
| **LEP^33^** | NM_001009850.1 | GACCTCTCGTCCCAAAGTCG | TGAGATCCTCAGGTCCGGTT | 59.96 | 253 |

^1^HPRT, hypoxanthine guanine phosphoribosyl transferase; ^2^RPL17, ribosomal protein L17; ^3^YWHAZ, tyrosine 3-monooxygenase/tryptophan 5-monooxygenase activation protein zeta polypeptide; ^4^ACLY, ATP Citrate lyase; ^5^ADIPOQ, Adiponectin, C1Q and collagen domain containing; ^6^ADIPOR1, Adiponectin receptor 1; ^7^FASN, Fatty acid synthase; ^8^SLC2A1, solute carrier family 2 member 1 (GLUT 1); ^9^SLC2A4, solute carrier family 2 member 4 (GLUT 4); ^10^FFAR4, G protein coupled receptor 120 / free fatty acid receptor 4; ^11^HCAR2, hydroxycarboxylic acid receptor 2; ^12^LIPE, Hormone sensitive lipase; ^13^INSR, Insulin receptor; ^14^IRS1, Insulin receptor substrate 1; ^15^IRS2, insulin receptor substrate 2; ^16^IFNG, interferon gamma; ^17^IL1B, Interleukin 1 beta; ^18^IL6, Interleukin 6; ^19^LPL, Lipoprotein Lipase; ^20^MDH1, cytosolic malate dehydrogenase; ^21^MDH2, Malate dehydrogenase 2 (mitochondrial); ^22^SERPINE1, plasminogen activator inhibitor 1; ^23^PPARG, Peroxisome proliferator activated receptor gamma 1; ^24^PIK3R1, phosphatidylinositol 3-kinase regulatory subunit alpha; ^25^RETN, Resistin; ^26^SREBF1, sterol regulatory binding protein-1c-like; ^27^TNF, Tumor necrosis factor; ^28^TLR4, Toll like receptor 4; ^29^SOCS5, suppressor of cytokine signaling 5; ^30^IL2RA, interleukin 2 receptor subunit alpha; ^31^CCR2, C-C motif chemokine receptor 2; ^32^CH2, major allergen I, polypeptide chain 2; ^33^LEP, Precursor Leptin.

**Suppl. Table 6.** Serum chemistry panel of cats supplemented with probiotic and postbiotic at day -30 (adaptation period).

|  |  | **Treatments** | | | |  | **p-value** |
| --- | --- | --- | --- | --- | --- | --- | --- |
| **Analytes** | **Reference range^2^** | | **CON** | **POST** | **PRO** | **SEM^1^** | **Trt** |
| **Creatinine(mg/dL)** | 0.5-1.5 | | 1.2 | 1.2 | 1.2 | 0.08 | 0.9883 |
| **BUN^3^ (mg/dL)** | 6.0-30.0 | | 20.0 | 19.7 | 19.4 | 1.02 | 0.9211 |
| **Total protein(g/dL)** | 5.1-7.0 | | 6.4 | 6.5 | 6.5 | 0.11 | 0.6781 |
| **Albumin(g/dL)** | 2.5-3.8 | | 2.9 | 2.8 | 2.8 | 0.05 | 0.173 |
| **Globulin(g/dL)** | 2.7-4.4 | | 3.5 | 3.7 | 3.7 | 0.12 | 0.2639 |
| **Alb/Glo ratio** | **------** | | 0.8 | 0.8 | 0.8 | 0.04 | 0.2092 |
| **Calcium(mg/dL)** | 7.6-11.4 | | 8.8 | 8.7 | 8.6 | 0.12 | 0.5399 |
| **Phosphorus(mg/dL)** | 2.7-5.2 | | 5.1 | 5.0 | 4.8 | 0.17 | 0.4861 |
| **Sodium(mmol/L)** | 141-152 | | 146.7 | 145.8 | 145.8 | 0.48 | 0.3373 |
| **Potassium(mmol/L)** | 3.9-5.5 | | 4.0 | 4.2 | 4.0 | 0.14 | 0.7105 |
| **Na/K ratio** | 28-36 | | 35.5 | 35.7 | 36.7 | 1.28 | 0.7929 |
| **Chloride(mmol/L)** | 107-118 | | 115.2 | 114.9 | 114.6 | 0.54 | 0.7511 |
| **Glucose(mg/dL)** | 68-126 | | 131.4 | 165.6 | 152.2 | 11.71 | 0.1312 |
| **Alkaline Phos t.** | 10-85 | | 22.3 | 20.3 | 18.5 | 1.73 | 0.3229 |
| **ALT^4^ (U/L)** | 14-71 | | 73.6 | 69.3 | 71.3 | 6.27 | 0.8917 |
| **CPK^5^ (U/L)** | 10-250 | | 108.3 | 93.2 | 99.2 | 8.41 | 0.4511 |
| **Cholesterol(mg/dL)** | 129-297 | | 108.9 | 103.7 | 93.2 | 4.95 | 0.0872 |
| **Triglycerides (mg/dL)** | 32-154 | | 42.5 | 41.1 | 37.1 | 2.95 | 0.3839 |
| **Bicarbonate (mmol/L)** | 16-24 | | 19.0 | 19.1 | 19.8 | 0.43 | 0.4106 |
| **Anion Gap** | 10-27 | | 16.6 | 15.9 | 15.6 | 0.36 | 0.4298 |
| **Fructosamine (μmol/L)** | 166 - 298 | | 230.2 | 216.6 | 228.2 | 6.26 | 0.2666 |

^1^SEM = Standard error of the mean

^2^Reference ranges were provided by the University of Illinois Veterinary Diagnostics Laboratory.

^3^BUN = Blood urea nitrogen, ^4^ALT = Alanine transaminase; ^5^CPK = Creatine phosphokinase

^x-y^Means within a row with different superscript tended to differ (P = 0.0872)

**Suppl. Table 7**. Complete blood count analysis of cats supplemented with probiotic and postbiotic at day -30 (adaptation period).

|  | **Treatments** | | |  |  |
| --- | --- | --- | --- | --- | --- |
| **Items/Day** | **CON** | **POST** | **PRO** | **SEM^1^** | **p-value** |
|  |  |  |  |  | **Trt** |
| **Red Blood Cells (x10^6/ul)** | 8.0 | 7.9 | 7.5 | 0.247 | 0.3360 |
| **Hemoglobin (g/dL)** | 11.6 | 11.5 | 10.9 | 0.343 | 0.3038 |
| **Hematocrit %** | 33.0 | 32.0 | 30.7 | 0.789 | 0.1306 |
| **Mean Cell Volume (fl)** | 41.6 | 41.5 | 41.2 | 0.749 | 0.9169 |
| **MCH (pg)** | 14.6 | 14.5 | 14.6 | 0.248 | 0.9591 |
| **MCHC (g/dL)** | 35.2 | 35.1 | 35.5 | 0.308 | 0.5596 |
| **Platelets** | 326.8 | 383.5 | 388.4 | 48.441 | 0.6082 |
| **White Blood Cell Count. (x10^3/ul)** | 13.2 | 12.1 | 12.9 | 0.934 | 0.6850 |
| **Lymph^2^ %.** | 36.4 | 28.6 | 31.9 | 2.980 | 0.1960 |
| **Mono^3^ %.** | 2.7 | 3.6 | 3.4 | 0.508 | 0.4288 |
| **Eos^4^ %.** | 4.8 | 6.2 | 5.6 | 0.683 | 0.3863 |
| **Baso^5^ %.** | 0.4 | 0.7 | 0.9 | 0.421 | 0.1904 |

^1^SEM = Standard error of the mean

^2^Lymph = lymphocytes; ^3^Mono = monocytes; ^4^Eos = Eosinophils; ^5^Baso = Basophils.

**Suppl.Table 8.** Serum cytokines and chemokines concentrations of cats supplemented with probiotic and postbiotic on day -30 (adaptation period).

|  | **Treatments** | | |  | | |
| --- | --- | --- | --- | --- | --- | --- |
| **Analytes** | **CON** | **POST** | **PRO** | | **SEM^1^** | **p-value** |
|  |  |  |  |  |  | **Trt** |
| **FAS^2^, pg/mL** | 12.9 | 27.5 | 18.3 | | 6.31 | 0.1836 |
| **Flt-3L^3^, pg/mL** | 89.6 | 70.0 | 84.3 | | 14.68 | 0.6230 |
| **GM-CSF^4^, pg/mL** | 8.7 | 19.0 | 19.5 | | 4.79 | 0.2221 |
| **IFN**$\boldsymbol{\gamma}$**^5^, pg/mL** | 216.2 | 249.0 | 229.3 | | 58.91 | 0.4344 |
| **IL-1**$\boldsymbol{\beta}$**^6^, pg/mL** | 35.1 | 37.6 | 58.1 | | 15.57 | 0.9299 |
| **IL-2^7^, pg/mL** | 23.8 | 40.8 | 37.5 | | 9.23 | 0.5952 |
| **PDGF-BB^8^, pg/mL** | 1478.3 | 1864.4 | 1744.6 | | 380.43 | 0.7660 |
| **IL-12(p40)^9^, pg/mL** | 620.9 | 395.3 | 462.5 | | 101.30 | 0.2840 |
| **IL-13^10^, pg/mL** | 14.9 | 28.1 | 23.2 | | 4.76 | 0.9722 |
| **IL-4^11^, pg/mL** | 415.1 | 490.4 | 962.2 | | 129.41 | 0.7624 |
| **IL-6^12^, pg/mL** | 222.3 | 222.6 | 444.0 | | 120.19 | 0.8281 |
| **IL-8^13^, pg/mL** | 54.2 | 39.0 | 53.3 | | 464.07 | 0.7637 |
| **KC^14^, pg/mL** | 13.6 | 12.3 | 22.4 | | 112.07 | 0.6005 |
| **SDF-1^15^, pg/mL** | 3524.3 | 2104.3 | 2433.5 | | 554.18 | 0.1813 |
| **RANTES^16^, pg/mL** | 68.0 | 47.6 | 52.3 | | 11.86 | 0.4540 |
| **SCF^17^, pg/mL** | 182.4 | 156.3 | 212.9 | | 49.42 | 0.7682 |
| **MCP-1^18^, pg/mL** | 1303.0 | 1728.9 | 1847.4 | | 281.67 | 0.3489 |
| **TNF**α**19, pg/mL** | 293.9 | 137.1 | 376.5 | | 125.43 | 0.2791 |
| **IL-18^20^, pg/mL** | 98.0 | 234.3 | 224.0 | | 60.27 | 0.2686 |

^1^SEM = Standard error of the mean

^2^FAS = Cell surface death receptor; ^3^ Flt-3L = Fms-related tyrosine kinase 3 ligand; ^4^GM-CSF = Granulocyte macrophage colony stimulating factor; ^5^IFN$\gamma$= Interferon gamma; ^6^IL-1$\beta$= Interleukin-1 beta; ^7^IL-2 = Interleukin-2; ^8^PDGF-BB = Platelet-derived growth factor-BB; ^9^IL-12 (p40) = Interleukin-12 (p40) subunit; ^10^IL-13 = Interleukin 13; ^11^IL-4 = Interleukin 4; ^12^IL-6 = Interleukin 6; ^13^IL-8 = Interleukin 8; ^14^KC = Keratinocyte chemoattractant; ^15^SDF-1 = Stromal cell-derived factor 1; ^16^RANTES = regulated on activation, normal T cell expressed and secreted; ^17^SCF = Stem cell factor; ^18^MCP-1 = Monocyte chemoattractant protein-1; ^19^TNFα = tumor necrosis factor alpha; ^20^IL-18 = Interleukin 18.

**Suppl. Table 9** Longitudinal assessment of serum chemistry of cats supplemented with probiotic and postbiotic.

|  |  | **Treatments** | | | | | | | | | | | |  |  | |  | |  | |
| --- | --- | --- | --- | --- | --- | --- | --- | --- | --- | --- | --- | --- | --- | --- | --- | --- | --- | --- | --- | --- |
|  |  | **CON** | | | | **POST** | | | | **PRO** | | | |  | **p-value** | | | | | |
|  | **Day** | **0** | **30** | **60** | **90** | **0** | **30** | **60** | **90** | **0** | **30** | **60** | **90** | **SEM^1^** | **Trt** | **Day** | | **Trt* Day** | |  |
| ***Analytes*** | **Ref. Range^2^** |  |  |  |  |  |  |  |  |  |  |  |  |  |  |  | |  | |  |
| **Creatinine (mg/dL)** | 0.5-1.5 | 1.2 | 1.2 | 1.2 | 1.2 | 1.2 | 1.2 | 1.2 | 1.2 | 1.2 | 1.2 | 1.1 | 1.1 | 0.08 | 0.8363 | 0.0011 | | 0.6442 | |  |
| **BUN**^3^ **(mg/dL)** | 6.0-30.0 | 18.3 | 19.3 | 19.3 | 19.9 | 19.5 | 20.4 | 19.5 | 21.0 | 19.3 | 19.2 | 19.0 | 19.9 | 0.86 | 0.7188 | 0.0006 | | 0.4806 | |  |
| **Total protein (g/dL)** | 5.1-7.0 | 6.8 | 6.7 | 6.4 | 6.5 | 6.8 | 6.8 | 6.5 | 6.8 | 6.8 | 6.8 | 6.4 | 6.6 | 0.11 | 0.7303 | 0.0001 | | 0.5328 | |  |
| **Albumin (g/dL)** | 2.5-3.8 | 3.0 | 3.0 | 3.1 | 3.1 | 2.9 | 2.9 | 3.0 | 3.0 | 2.8 | 2.9 | 3.0 | 2.9 | 0.07 | 0.2540 | 0.0001 | | 0.6165 | |  |
| **Globulin (g/dL)** | 2.7-4.4 | 3.8 | 3.7 | 3.3 | 3.5 | 3.9 | 3.9 | 3.5 | 3.7 | 4.0 | 3.9 | 3.5 | 3.7 | 0.12 | 0.3707 | 0.0001 | | 0.8320 | |  |
| **Alb/Glo ratio** | **-----** | 0.8 | 0.8 | 1.0 | 0.9 | 0.7 | 0.8 | 0.9 | 0.8 | 0.7 | 0.8 | 0.9 | 0.8 | 0.04 | 0.2509 | 0.0001 | | 0.4208 | |  |
| **Calcium (mg/dL)** | 7.6-11.4 | 8.9 | 8.9 | 8.9 | 8.7 | 9.0 | 8.8 | 8.8 | 8.6 | 8.8 | 8.7 | 8.5 | 8.5 | 0.15 | 0.4775 | 0.0001 | | 0.4354 | |  |
| **Phosphorus (mg/dL)** | 2.7-5.2 | 4.9 | 4.8 | 5.1 | 5.0 | 4.8 | 4.8 | 4.8 | 5.2 | 4.5 | 4.7 | 4.8 | 4.5 | 0.17 | 0.2341 | 0.4010 | | 0.2129 | |  |
| **Sodium (mmol/L)** | 141-152 | 147.1 | 148.1 | 148.3 | 147.7 | 147.2 | 148.1 | 147.6 | 147.2 | 147.2 | 147.6 | 147.2 | 147.1 | 0.15 | 0.5435 | 0.0462 | | 0.7263 | |  |
| **Potassium (mmol/L)** | 3.9-5.5 | 3.8 | 4.0 | 3.9 | 4.1 | 4.2 | 3.9 | 4.1 | 4.2 | 4.2 | 4.0 | 4.1 | 4.1 | 0.15 | 0.7419 | 0.0803 | | 0.2472 | |  |
| **Na/K ratio** | 28-36 | 39.2 | 37.8 | 39.0 | 36.6 | 35.8 | 39.0 | 36.7 | 35.2 | 35.8 | 37.4 | 37.9 | 36.4 | 1.64 | 0.7194 | 0.0792 | | 0.3070 | |  |
| **Chloride (mmol/L)** | 107-118 | 114.9 | 116.2 | 116.2 | 115.3 | 114.7 | 116.5 | 116.5 | 115.0 | 114.1 | 115.8 | 116.6 | 114.8 | 1.42 | 0.8742 | 0.0001 | | 0.1327 | |  |

^1^SEM = Standard error of the mean.

^2^Reference ranges were provided by the University of Illinois Veterinary Diagnostics Laboratory.

^3^BUN = Blood urea nitrogen

**Suppl. Table 9(cont.).** Longitudinal assessment of serum chemistry of cats supplemented with probiotic and postbiotic.

|  |  | **Treatments** | | | | | | | | | | | |  |  |  |  |
| --- | --- | --- | --- | --- | --- | --- | --- | --- | --- | --- | --- | --- | --- | --- | --- | --- | --- |
|  |  | **CON** | | | | **POST** | | | | **PRO** | | | |  | **p-value** | | |
|  | **Day** | **0** | **30** | **60** | **90** | **0** | **30** | **60** | **90** | **0** | **30** | **60** | **90** | **SEM^1^** | **Trt** | **Day** | **Trt* Day** |
| ***Analytes*** | **Reference range^2^** | |  |  |  |  |  |  |  |  |  |  |  |  |  |  |  |
| **Glucose (mg/dL)** | 68-126 | 120.3 | 138.7 | 118.3 | 128.1 | 165.0 | 137.5 | 133.1 | 147.8 | 153.3 | 140.2 | 130.5 | 165.7 | 12.51 | 0.2009 | 0.0612 | 0.2287 |
| **Alkaline Phos t. (U/L)** | 10-85 | 22.7 | 24.1 | 23.9 | 24.3 | 19.9 | 20.9 | 21.7 | 23.4 | 19.0 | 19.7 | 19.0 | 19.8 | 4.22 | 0.4826 | 0.0073 | 0.4991 |
| **ALT^3^ (U/L)** | 14-71 | 55.8 | 53.7 | 51.8 | 59.0 | 62.9 | 54.0 | 55.5 | 55.6 | 61.8 | 49.3 | 53.6 | 54.6 | 3.84 | 0.866 | 0.0035 | 0.3371 |
| **CPK^4^ (U/L)** | 10-250 | 108.4 | 107.0 | 99.8 | 115.7 | 115.8 | 110.4 | 118.4 | 112.2 | 94.7 | 100.2 | 90.9 | 99.3 | 8.44 | 0.1986 | 0.6522 | 0.5613 |
| **Cholesterol (mg/dL)** | 129-297 | 111.3 | 117.4 | 118.6 | 127.0 | 105.6 | 112.6 | 111.6 | 115.5 | 104.4 | 102.7 | 110.1 | 108.8 | 3.84 | 0.2722 | 0.0009 | 0.1066 |
| **Triglycerides (mg/dL)** | 32-154 | 40.8 | 44.8 | 44.4 | 47.2 | 43.0 | 45.3 | 44.7 | 53.6 | 40.0 | 37.6 | 40.8 | 40.3 | 4.70 | 0.5009 | 0.0386 | 0.4316 |
| **Bicarbonate (mmol/L)** | 16-24 | 19.9^a^ | 18.5^b^ | 18.8^ab^ | 19.0^ab^ | 19.7^ab^ | 18.7^ab^ | 18.6^ab^ | 18.8^ab^ | 18.9^ab^ | 19.6^ab^ | 18.7^ab^ | 20.0^a^ | 0.41 | 0.7768 | 0.0009 | **0.0002** |
| **Anion Gap** | 10-27 | 16.2^b^ | 17.7^a^ | 17.2^ab^ | 17.5^ab^ | 17.0^ab^ | 17.0^ab^ | 16.7^ab^ | 17.7^ab^ | 18.4^a^ | 16.3^b^ | 15.9^b^ | 16.3^b^ | 0.40 | 0.4982 | 0.0949 | **0.0001** |
| **Fructosamine (μmol/L)** | 166-298 | 230.2 | 237.4 | 240.7 | 235.0 | 214.7 | 224.1 | 229.6 | 228.3 | 228.2 | 221.5 | 227.4 | 220.7 | 6.65 | 0.3215 | 0.0069 | 0.1242 |

^1^SEM = Standard error of the mean.

^2^Reference ranges were provided by the University of Illinois Veterinary Diagnostics Laboratory.

^3^ALT = Alanine transaminase; ^4^CPK = Creatine phosphokinase

^a-b^ Means within a row with different superscript letters are different (P < 0.05).

**Suppl. Table 10.** Longitudinal assessment of serum leptin and oxidative biomarker concentrations of cats supplemented with probiotic and postbiotic.

|  | **Treatments** | | | | | | | | | | | |  |  | | |
| --- | --- | --- | --- | --- | --- | --- | --- | --- | --- | --- | --- | --- | --- | --- | --- | --- |
|  | **CON** | | | | **POST** | | | | **PRO** | | | |  | **p-value** | | |
| **Analytes/ Day** | **0** | **30** | **60** | **90** | **0** | **30** | **60** | **90** | **0** | **30** | **60** | **90** | **SEM^1^** | **Trt** | **Day** | **Trt*Day** |
| **Leptin, ng/ml** | 0.9 | 1.0 | 1.3 | 2.1 | 0.8 | 1.0 | 1.4 | 1.8 | 0.8 | 0.8 | 1.4 | 1.8 | 0.17 | 0.6659 | 0.0001 | 0.9055 |
| **MDA^2^, μmol/L** | 45.2 | 67.1 | 41.1 | 28.0 | 55.7 | 65.1 | 29.9 | 26.6 | 44.2 | 63.7 | 35.3 | 26.3 | 5.74 | 0.7612 | 0.0001 | 0.6742 |
| **SOD^3^, ng/ml** | 0.3 | 0.4 | 0.7 | 0.7 | 0.2 | 0.3 | 0.7 | 0.1 | 0.2 | 0.5 | 0.9 | 0.3 | 0.14 | 0.2860 | 0.0001 | 0.2884 |

^1^SEM = Standard error of the mean.

^2^MDA (μmol/L) = Malondialdehyde; ^3^SOD (ng/ml) = Superoxide dismutase

**Suppl. Table 11**. Longitudinal assessment of complete blood cell count analysis of cats supplemented with probiotic and postbiotic.

|  |  | Treatment | | | | | | | | | | | |  |  |  |  |
| --- | --- | --- | --- | --- | --- | --- | --- | --- | --- | --- | --- | --- | --- | --- | --- | --- | --- |
|  |  | CON | | | | POST | | | | PRO | | | |  | p-value | | |
| **Items/Day** | **Reference range** | **0** | **30** | **60** | **90** | **0** | **30** | **60** | **90** | **0** | **30** | **60** | **90** | **SEM^1^** | **Trt** | **Day** | **Trt*Day** |
| **Red Blood Cells (x10^6/ul)** | 5 - 10 | 8.8 | 8.5 | 8.8 | 8.5 | 8.2 | 8.2 | 8.3 | 8.5 | 8.2 | 8.0 | 8.1 | 8.2 | 0.287 | 0.3343 | 0.3705 | 0.7026 |
| **Hemoglobin (g/dL)** | 8 - 15 | 12.2 | 11.8 | 12.1 | 11.7 | 11.4 | 11.3 | 11.3 | 11.4 | 11.3 | 11.0 | 11.0 | 10.9 | 0.319 | **0.0746** | 0.2856 | 0.8605 |
| **Hematocrit %** | 30 - 45 | 35.6 | 34.0 | 35.1 | 34.3 | 33.0 | 32.9 | 32.7 | 33.2 | 32.7 | 31.5 | 31.7 | 31.9 | 0.841 | **0.0148** | 0.2616 | 0.8448 |
| **Mean Cell Volume (fl)** | 37 - 55 | 40.5 | 40.3 | 40.2 | 40.3 | 40.5 | 40.2 | 39.5 | 39.5 | 40.1 | 39.6 | 39.2 | 39.1 | 0.823 | 0.7731 | 0.0001 | 0.1847 |
| **MCH^2^ (pg)** | 13 - 18 | 13.9 | 13.9 | 13.8 | 13.7 | 14.0 | 13.9 | 13.6 | 13.5 | 13.8 | 13.8 | 13.6 | 13.4 | 0.258 | 0.8874 | 0.0001 | 0.2679 |
| **MCHC^3^ (g/dL)** | 29 - 38 | 34.2 | 34.6 | 34.4 | 34.0 | 34.5 | 34.5 | 34.5 | 34.3 | 34.4 | 34.9 | 34.7 | 34.3 | 0.325 | 0.8329 | 0.0004 | 0.6900 |
| **Platelets** | 300 - 700 | 417.9 | 363.3 | 466.4 | 388.6 | 364.1 | 369.7 | 499.8 | 594.5 | 374.0 | 398.4 | 481.7 | 464.6 | 48.912 | 0.4090 | 0.0069 | 0.2910 |
| *x10^3/ul* |  |  |  |  |  |  |  |  |  |  |  |  |  |  |  |  |  |
| **White Blood Cell Count** | 5.5 -19.5 | 14.6 | 13.2 | 11.4 | 12.4 | 12.8 | 11.5 | 10.1 | 11.4 | 12.4 | 12.1 | 11.0 | 11.8 | 1.066 | 0.5528 | 0.0001 | 0.5765 |
| **A Lymphocytes** | 1.7 - 7 | 4.6 | 4.7 | 3.5 | 3.9 | 3.7 | 3.0 | 3.5 | 3.4 | 3.7 | 3.7 | 2.9 | 3.1 | 0.399 | 0.1673 | 0.0059 | 0.2034 |
| **A Monocytes** | 0 -.9 | 0.4 | 0.4 | 0.3 | 0.3 | 0.4 | 0.3 | 0.4 | 0.4 | 0.4 | 0.3 | 0.3 | 0.4 | 0.055 | 0.7698 | 0.1223 | 0.7513 |
| **A Eosinophils** | 0 - 0.8 | 0.8 | 0.6 | 0.7 | 0.6 | 0.9 | 0.8 | 0.6 | 0.8 | 0.8 | 0.8 | 0.6 | 0.8 | 0.107 | 0.7067 | 0.0133 | 0.1483 |
| **A Basophils** | 0 .2 | 0.1 | 0.1 | 0.0 | 0.0 | 0.1 | 0.1 | 0.0 | 0.1 | 0.1 | 0.1 | 0.1 | 0.1 | 0.037 | 0.5251 | 0.3638 | 0.3525 |
| **Lymphocytes %.** |  | 31.7 | 36.9 | 32.2 | 32.8 | 29.0 | 26.3 | 29.8 | 29.5 | 30.7 | 31.5 | 28.2 | 27.5 | 2.829 | 0.3248 | 0.6737 | 0.1820 |
| **Monocytes %.** |  | 3.0 | 2.9 | 2.4 | 2.4 | 3.3 | 2.1 | 3.7 | 3.2 | 3.1 | 3.0 | 2.6 | 3.2 | 0.407 | 0.4478 | 0.5345 | 0.1319 |
| **Eosinophils %.** |  | 5.7 | 4.6 | 6.4 | 4.5 | 7.3 | 7.6 | 6.1 | 7.2 | 6.9 | 6.6 | 5.8 | 6.7 | 0.872 | 0.2012 | 0.7329 | 0.1319 |
| **Basophils %.** |  | 0.7 | 0.4 | 0.3 | 0.3 | 0.7 | 0.8 | 0.3 | 1.0 | 0.4 | 0.3 | 0.4 | 0.7 | 0.269 | 0.3896 | 0.5209 | 0.4068 |

^1^SEM = Standard error of the mean

^2^MCH = mean corpuscular hemoglobin; ^3^MCHC = mean corpuscular hemoglobin concentration.

**Suppl. Table 12**. Fold change of selected genes, in adult cats supplemented with probiotic and postbiotic.

|  | **Treatments** | | | | | | | | | | | |  |  |  |  | |
| --- | --- | --- | --- | --- | --- | --- | --- | --- | --- | --- | --- | --- | --- | --- | --- | --- | --- |
|  | **CON** | | | | **POST** | | | | **PRO** | | | | **SEM^1^** | **p-value** | | | |
| **Items/Day** | **0** | **30** | **60** | **90** | **0** | **30** | **60** | **90** | **0** | **30** | **60** | **90** | **Trt** | **Trt** | **Day** | **Trt*Day** |  |
| **YWHAZ^2^** | 1.02 | 1.29 | 0.99 | 1.19 | 1.00 | 0.96 | 0.97 | 0.84 | 0.98 | 0.99 | 0.99 | 0.90 | 0.1276 | 0.4968 | 0.4503 | 0.7202 |  |
| **ACLY^3^** | 1.02 | 1.19 | 0.82 | 1.06 | 1.00 | 0.95 | 0.94 | 0.73 | 0.98 | 0.91 | 0.84 | 0.87 | 0.1135 | 0.3747 | 0.1604 | 0.3706 |  |
| **ADIPOQ^4^** | 1.02 | 1.06 | 0.95 | 1.43 | 1.00 | 2.08 | 1.14 | 0.86 | 0.98 | 0.93 | 0.72 | 0.73 | 0.2399 | 0.1996 | 0.3075 | 0.3847 |  |
| **ADIPOR1^5^** | 1.00 | 1.00 | 1.18 | 1.49 | 1.00 | 2.52 | 1.79 | 2.01 | 1.00 | 2.25 | 1.12 | 1.33 | 0.6416 | 0.9653 | 0.5323 | 0.8107 |  |
| **FASN^6^** | 1.04 | 0.90 | 0.77 | 1.00 | 1.00 | 0.94 | 0.90 | 0.70 | 0.96 | 0.84 | 0.71 | 0.86 | 0.1418 | 0.7919 | 0.1986 | 0.7303 |  |
| **HCAR2^7^** | 1.07 | 1.93 | 1.05 | 1.08 | 1.00 | 0.75 | 0.84 | 1.48 | 0.93 | 0.88 | 0.76 | 0.99 | 0.2769 | 0.7747 | 0.4800 | 0.4021 |  |
| **LIPE^8^** | 1.00 | 1.02 | 0.75 | 1.33 | 1.00 | 1.02 | 0.90 | 0.93 | 1.00 | 1.01 | 0.83 | 0.93 | 0.1557 | 0.7381 | 0.2767 | 0.6363 |  |
| **INSR^9^** | 1.11 | 1.73 | 1.64 | 2.78 | 1.00 | 0.81 | 1.02 | 1.41 | 0.89 | 0.85 | 1.17 | 1.24 | 0.6311 | 0.2244 | 0.1879 | 0.6620 |  |
| **IRS1^10^** | 1.01 | 1.51 | 0.78 | 0.73 | 1.00 | 1.04 | 0.90 | 0.77 | 0.99 | 0.98 | 0.76 | 0.69 | 0.8299 | 0.3882 | 0.0007 | 0.3251 |  |
| **IRS2^11^** | 1.04 | 1.18 | 0.80 | 1.11 | 1.00 | 0.94 | 0.92 | 0.77 | 0.96 | 0.87 | 0.71 | 0.78 | 0.8829 | 0.3482 | 0.2306 | 0.7288 |  |
| **INFG^12^** | 1.00 | 1.69 | 1.51 | 1.85 | 1.00 | 1.58 | 1.21 | 2.21 | 1.00 | 0.98 | 1.31 | 1.49 | 1.0094 | 0.3810 | 0.9722 | 0.5302 |  |
| **IL1B^13^** | 1.03 | 1.31 | 0.74 | 0.80 | 1.00 | 0.69 | 0.67 | 0.66 | 0.97 | 0.79 | 0.62 | 0.72 | 0.1398 | 0.1606 | 0.0046 | 0.2748 |  |
| **MDH1^14^** | 1.02 | 1.06 | 0.96 | 0.97 | 1.00 | 0.98 | 1.01 | 0.85 | 0.98 | 1.01 | 0.80 | 0.97 | 0.1017 | 0.7575 | 0.4877 | 0.7655 |  |
| **MDH2^15^** | 1.07 | 1.40 | 1.01 | 1.15 | 1.00 | 0.84 | 0.77 | 0.88 | 0.93 | 0.78 | 0.82 | 0.83 | 0.1664 | 0.2981 | 0.0792 | 0.8987 |  |
| **SERPINE1^16^** | 1.00 | 1.14 | 0.69 | 0.86 | 1.00 | 1.00 | 0.83 | 1.67 | 1.00 | 1.30 | 1.00 | 1.62 | 0.2901 | 0.3455 | 0.2191 | 0.3113 |  |
| **PPARG^17^** | 1.16 | 1.86 | 2.05 | 1.60 | 1.00 | 0.97 | 1.53 | 1.80 | 0.84 | 1.06 | 1.19 | 1.33 | 0.4321 | 0.2298 | 0.3273 | 0.3814 |  |
| **PIK3R1^18^** | 1.02 | 1.09 | 0.85 | 1.00 | 1.00 | 0.90 | 0.89 | 0.83 | 0.98 | 0.92 | 0.76 | 0.87 | 0.1097 | 0.5022 | 0.1594 | 0.9158 |  |
| **TNF^19^** | 1.03 | 1.04 | 0.82 | 0.91 | 1.00 | 0.89 | 0.88 | 0.77 | 0.97 | 0.99 | 0.81 | 0.93 | 0.1202 | 0.8221 | 0.2073 | 0.9381 |  |
| **TLR4^20^** | 1.01 | 1.32 | 0.79 | 0.85 | 1.00 | 0.79 | 0.82 | 0.58 | 0.99 | 0.81 | 0.82 | 0.84 | 0.1393 | 0.2312 | 0.0633 | 0.2053 |  |
| **IL2RA^22^** | 1.03 | 1.50 | 1.23 | 1.62 | 1.00 | 1.14 | 1.29 | 0.84 | 0.97 | 1.01 | 0.97 | 1.04 | 0.2054 | 0.3913 | 0.9115 | 0.1689 |  |
| **CH2^24^** | 1.00 | 0.74 | 1.63 | 2.23 | 1.00 | 0.94 | 0.80 | 2.00 | 1.00 | 2.52 | 1.01 | 1.49 | 0.8967 | 0.8781 | 0.3263 | 0.2362 |  |
| **LEP^25^** | 0.99 | 0.68 | 1.17 | 2.09 | 1.00 | 1.12 | 1.46 | 2.14 | 1.01 | 1.29 | 0.92 | 1.28 | 0.4256 | 0.3363 | 0.3987 | 0.3702 |  |

^1^SEM = Standard error of the mean

^2^YWHAZ, tyrosine 3-monooxygenase/tryptophan 5-monooxygenase activation protein zeta polypeptide; ^3^ACLY, ATP Citrate lyase; ^4^ADIPOQ, Adiponectin, C1Q and collagen domain containing; ^5^ADIPOR1, Adiponectin receptor 1; ^6^FASN, Fatty acid synthase; ^7^HCAR2, hydroxycarboxylic acid receptor 2; ^8^LIPE, Hormone sensitive lipase; ^9^INSR, Insulin receptor; ^10^IRS1, Insulin receptor substrate 1; ^11^IRS2, insulin receptor substrate 2; ^13^IL1B, Interleukin 1 beta; ^14^MDH1, cytosolic malate dehydrogenase; ^15^MDH2, Malate dehydrogenase 2 (mitochondrial); ^16^SERPINE1, plasminogen activator inhibitor 1; ^17^PPARG, Peroxisome proliferator activated receptor gamma 1; ^18^PIK3R1, phosphatidylinositol 3-kinase regulatory subunit alpha; ^19^TNF, Tumor necrosis factor; ^20^TLR4, Toll like receptor 4; ^21^SOCS5, suppressor of cytokine signaling 5; ^22^IL2RA, interleukin 2 receptor subunit alpha; ^23^CCR2, C-C motif chemokine receptor 2; . ^24^CH2, major allergen I, polypeptide chain 2; ^25^LEP, precursor leptin.
